# Biomarker development in MET-targeted therapy

**DOI:** 10.18632/oncotarget.8276

**Published:** 2016-03-22

**Authors:** Yanni Zhang, Zhiqiang Du, Mingqiang Zhang

**Affiliations:** ^1^ Amgen Biopharmaceutical Research and Development (Shanghai) Co., Ltd, Shanghai, China

**Keywords:** MET, amplification, overexpression, biomarker, MET-targeted therapy

## Abstract

Activation of the MET receptor tyrosine kinase by its ligand, hepatocyte growth factor (HGF), has been implicated in a variety of cellular processes, including cell proliferation, survival, migration, motility and invasion, all of which may be enhanced in human cancers. Aberrantly activated MET/HGF signaling correlates with tumorigenesis and metastasis, and is regarded as a robust target for the development of novel anti-cancer treatments. Various clinical trials were conducted to evaluate the safety and efficacy of selective HGF/MET inhibitors in cancer patients. There is currently no optimal or standardized method for accurate and reliable assessment of MET levels, or other biomarkers that are predictive of the patient response to MET-targeted therapeutics. In this review, we discuss the importance of accurate HGF/MET signal detection as a predictive biomarker to guide patient selection for clinical trials of MET-targeted therapies in human cancers.

## INTRODUCTION

Receptor tyrosine kinases (RTKs) are involved in many vital processes including mammalian development, cell function and tissue homeostasis. The RTK MET and its ligand hepatocyte growth factor (HGF) activate multiple signaling pathways mediating embryogenesis, tissue regeneration and wound repair under normal physiological conditions [1]. However, an aberrant MET/HGF axis results in cell migration and survival and promotes tumor development and progression [2–4].

The MET proto-oncogene, located on chromosome 7 (7q21-31), is widely expressed in the epithelial cells of organs such as the liver, lung, gastrointestinal tract, and kidney during embryogenesis and adulthood [5]. HGF binding to the extracellular domain of MET results in its homodimerization and autophosphorylation at multiple tyrosine residues, including Y1234 and Y1235 in the kinase domain and subsequently Y1349 and Y1356 in the carboxy-terminal tail [6, 7]. Tyrosine phosphorylation of MET results in its activation and recruitment of signaling effectors, including adaptor proteins Grb2, Gab-1, Src and SHC, and subsequent phosphorylation of downstream transducers such as PI3K, ERK, PLC-γ, FAK and STAT3 [8]. HGF-induced MET activation can trigger cell proliferation, survival, motility, invasion, angiogenesis and branching morphogenesis [9–11].

Gab-1 binding to MET through GRB2 primarily leads to activation of the phosphoinositide 3-kinase (PI3K)/Akt and mitogen-activated protein kinase (MAPK)/ERK pathways. In the MAPK cascade, MET activation stimulates SHC and GRB2 recruitment to Gab-1, leading to MAPK activation [12], along with recruitment of SHP2 to Gab-1 that can link MET signaling to the MAPK cascade and extend the duration of MAPK phosphorylation [13]. The ERK-MAPK pathway is responsible for cell proliferation, cell-cycle progression and cell motility. The p85 subunit of PI3K can bind to MET directly and signal through the AKT pathway, stimulating cell survival [14]. Additional pathways responsible for cell migration and invasion response to MET signaling including RAS, CRK, and focal adhesion kinase (FAK) signaling [15]. STAT3 activation and nuclear translocation following MET binding is associated with tissue invasion [16]. MET ubiquitination by E3-ubiquitin ligase c-Cbl leads to MET degradation and is critical for controlled regulation of MET activity [17].

Interactive crosstalk between MET and other cell membrane proteins has been heavily investigated due to the development of drug resistance in targeted therapies. For example, the adhesion protein CD44 variant (CD44v6) is thought to promote downstream activation of the Ras pathway by complex formation between GRB2 and ezrin, radixin and moesin [18]. MET can bind integrin α6β4 in the presence of HGF, resulting in integrin phosphorylation and cell invasion. MET can be transactivated by directly binding epidermal growth factor receptor (EGFR) in the absence of HGF and presence of EGF or TGF-α [19]. Interactions between MET and other RTKs, including RON, PDGFR, Axl, HER2, ERBB3 and VEGFR family members, have been observed in a variety of cancers [20, 21].

### Aberrant MET activation in cancer

Aberrant HGF/MET axis activation has been implicated in the progression of multiple human tumor types, including liver, lung and gastric carcinomas [22, 23], and results in cell survival and migration and tumor development and progression [2–4]. HGF/MET signaling pathway activation can occur *via MET* gene amplification [24], overexpression [25], mutations [26–28] or paracrine and autocrine activation of MET by HGF [29], all of which have been observed in multiple human tumor types [22, 23].

MET overexpression has been reported in many human cancers, such as hepatocellular carcinoma (HCC) and non-small cell lung cancer (NSCLC), and correlates with poor prognosis. MET overexpression can occur *via*: 1) other tumor growth factors, such as EGF and interleukin-1 [25]; 2) transcription regulation by HIF-1α caused by hypoxia in the growing tumor [30]; 3) deregulation by transcription factors Ets and Sp1; or 4) downregulation of microRNAs targeting MET, such as miR-34 or miR-199a-3p [31–33].

*MET* gene amplification resulting in protein overexpression and constitutive activation of the MET receptor has been described in NSCLC, gastric carcinoma and HCC, as well as in preclinical models [24] ‘addicted’ to the MET signaling pathway. In gastric cancer, MET activation has been attributed to *MET* gene amplification or overexpression, which reduces apoptosis and promotes tumor cell survival, proliferation, differentiation and migration [34, 35].

*MET* mutations occur only rarely in cancers, but may correlate with tumor development. Constitutively activated MET mutations alter the molecular conformation of the protein structure, either promoting receptor dimerization or modifying catalytic activity [15]. Missense mutations in MET tyrosine kinase domains were recently detected in hereditary papillary renal cell carcinoma (RCC) [26], childhood HCC [27] and other cancers, and these residues were speculated to inhibit MET enzymatic activity. Somatic mutations have been observed in the MET juxtamembrane domain, deleting the exon responsible for E3-ubquitin protein ligase Cbl recruitment and reducing MET degradation [28]. Additional mutations have been identified in the MET sema domain in lung cancer, and are associated with HGF binding and receptor dimerization.

## MET AS A PREDICTIVE CANCER BIOMARKER

MET status in patients may serve as a potential biomarker for disease prognosis and a predictor of response to HGF/ MET inhibitors in the clinic. Tables 1, 2 and 3 summarize *MET* gene and protein expression patterns reported from different platforms in gastric, lung and liver carcinomas, respectively. Different reagents and scoring systems that define clinical MET positivity, and correlations between MET status and patient prognosis or outcome are discussed.

**Table 1:** Molecular alterations of MET/HGF in human gastric cancer

Alteration	Findings	Population	Technique	Evaluation	Reference
Amplification	15 (23%) of the 64 advanced gastric carcinomas showed the *c-MET* gene amplification	Japan	Southern blot	Amplification of the *c-MET* gene was defined as 3-fold or more increase of signal intensities than those of the corresponding non-neoplastic mucosa by densitometry tracing	[95]Kuniyasu et al., 1992
Amplification	*c-MET* amplification in 10% (7/70) of patients with primary gastric cancer	Japan	Slot Blot Hybridization	Fold amplification of the *c-MET* gene relative to each normal mucosa	[36]Tsugawa et al., 1998
Amplification	*MET* amplification in 10.2% of 128 primary gastric carcinoma patients without chemotherapy	Japan	Southern blot	Comparing the levels of *MET* gene in tumor tissue with those in the respective normal gastric mucosa	[53]Nakajima et al., 1999
Amplification	21.2% of FFPE primary tumor tissues from 472 patients had a *MET* copy number greater than 4.0 copies	Korea	qPCR	*MET* copy number >4.0 copies defined as *MET* amplification	[37]Lee et al., 2011
Amplification	0/38 patients with locally advanced gastric cancer	US	FISH	*MET* amplification defined as *MET*/*CEP7* ratio > 2	[54]Janjigian et al., 2011
Amplification	In 216 assessable patients, *MET* CNG five or more copies occurred in 21 patients (10%) with stage II or III radically resected gastric cancer	Italy	qPCR	CNG ≥5 copies as *MET* positive	[96]Graziano et al., 2011
Amplification	*MET* amplification in ten (2%) of 489 patients with GEC	Boston	FISH	Gene amplification as a gene-to-CN control probe ratio G:CN > 2.2 scored in 50 tumor nuclei	[38]Lennerz et al., 2011
Amplification	High polysomy of chromosome 7 and GA in 61 (16.0%) and 13 (3.4%) of 381 primary gastric carcinoma patients	Seoul, Korea	1. SISH 2. qPCR	1. Positive SISH: high polysomy (≥4 copies in ≥40% of cells; GA (defined by the presence of tight *MET* gene clusters and a ratio of *MET* gene to-chromosome of ≥2 or ≥15 copies of *MET* per cell in ≥10% of analyzed cells 2. Normalized gene ratios were interpreted as follows: <2=negative for GA and ≥2.0=positive for GA. All results were normalized vs respective amounts of *RNaseP* DNA (Applied Biosystems)	[40]Lee et al., 2012
Amplification	*MET* amplification observed in 8.3% (19/230 cases) with recurrent/Metastatic GC after chemotherapy	Guangzhou, China	FISH	FISH+ (GA): *MET*/*CEP7* ≥2 or ≥15 copies of *MET* per cell in ≥10% of analyzed cells	[39]An et al., 2013
Amplification	*MET* amplification in 4 of 266 FFPE specimens (1.5%) of advanced gastric cancer	Japan	1. qPCR 2. FISH	1. CNG > 4 copies as *MET* positive 2. Gene amplification defined as a mean *MET*/*CEN7p* copy number ratio of >2.2	[97]Kawakami et al., 2013
Amplification	In 95 patients with advanced GC treated with chemotherapy, 15 (16%) *MET* CNG>=5 copies cases	Italy	qPCR	Ct value for the copy number and reference assay was imported into the CopyCaller Software (Applied Biosystems) for post-PCR data analysis; CNG ≥5 copies (*MET*+)	[98]Graziano et al., 2014
Amplification	*MET* amplifications in 12 (6.1%) of 196 GC patients	Shanghai, China	FISH	For MET analysis, tumors with *MET* to *CEP7* ≥2 or presence of ≥10% gene cluster were defined as amplified	[41]Liu et al., 2014
Point mutation	Juxtamembrane domain: 1% (1/85) patients with primary gastric cancer	Korea	1. DHPLC 2. cold SSCP	gastric carcinoma DNA compared to normal gastric tissue DNA	[49]Lee et al., 2000
Overexpression	MET overexpression: 46.1% (59/128 patients with primary gastric carcinoma and without chemotherapy	Japan	IHC	Tumors that were stained positively for membrane and cytoplasm were considered to be positive for the expression of the c-MET. Only distinct staining in more than 5% of tumor cells was recorded as positive immunoreactivity	[53]Nakajima et al., 1999
Overexpression	In the IHC study, c-MET overexpression in (71.1%) 32 of 45 patients in gastric carcinoma compared with matched normal gastric tissues	Taiwan	IHC	The tumors were considered as positive immunreactivities if ≥ 5% of neoplastic cells showed distinct plasma membrane staining	[99]Huang et al., 2001
Overexpression	MET overexpression in 63% of 38 patients with locally advanced gastric cancer	US	IHC	The percentage of positive tumor cells (scale 0%–100%) with staining intensity from 0 to 3+. Positive IHC expression is defined as 25% or more staining with intensity 2 or 3+	[54]Janjigian et al., 2011
Overexpression	MET protein expression: 104 (23.7%) of 438 patients with primary gastric carcinoma,94 (21.5%) with IHC 2+ and 10 (2.3%) cases with IHC 3+	Seoul, Korea	IHC	No membrane staining or membrane staining in <10% of tumour cells (score 0), faint/barely perceptible partial membrane staining in > 10% of tumour cells (score 1+), weak-to-moderate staining of the entire membrane in > 10% of tumour cells (score 2+), and strong staining of the entire membrane in > 10% of tumour cells (score 3+). Scores of 0 and 1+ were considered as negative for MET overexpression, and scores of 2+ and 3+ were considered as positive	[40]Lee et al., 2012
Overexpression	MET overexpression in 108 (21.8%) of 495 patients in advanced gastric carcinoma	Korea	IHC	Both membranous and cytoplasmic staining was scored as follows: 0, no reactivity or faint staining; 1+, faint or weak staining; 2+, moderate staining; 3+, strong staining in >10% of tumor cells. MET overexpression was defined as 2+ or 3+ by membranous and cytoplasmic interpretation	[55]Ha et al., 2013
Overexpression	MET overexpression (IHC3+) in 9.6% (22/229 cases) with recurrent/Metastatic GC after chemotherapy	Guangzhou, China	IHC	The staining intensity and percentage of positive cells were assessed: 3+, ≥ 50% tumor cells with strong membrane/cytoplasm staining; 2+, ≥50% of tumor cells with moderate membrane/cytoplasm staining; 1+, ≥50% of tumor cells with weak membrane/cytoplasm staining; 0, No staining or ≤50% of tumor cells with membrane/cytoplasm staining of any intensity;	[39]An et al., 2013
Overexpression	MET overexpression (IHC2+ or 3+) in 12.3% (26/212 cases) of GC patients	Shanghai, China	IHC	DAKO HercepTest guideline used to semi-quantitatively score MET expression. MET+ defined by IHC2+/IHC3+	[41]Liu et al., 2014

**Table 2:** Molecular alterations of MET/HGF in human NSCLC

Alteration	Findings	Population	Technique	Evaluation	Reference
Point mutation	*MET* a somatic exon 14 deleting splice variant in 3 (1.7%) of 178 NSCLC samples	Japan	PCR-based sequencing	The sequencing of cDNA from tumor tissues compared to adjacent normal lung tissues	[51]Okuda et al., 2008
Point mutation	*MET* mutations in exon 14 in 4% of NSCLC patients	Italy	PCR-based sequencing	DNA sequence compared with wild-type nucleotide sequence	[52]Ludovini et al., 2012
Amplification	*MET* amplification in 4 of 18 (22%) *EGFR* mutant NSCLCs that had developed resistance to gefitinib or erlotinib	U.S.A	1. qPCR 2. FISH	1. relative to a reference, the Line-1 repetitive element: *MET* copy number ≥4 (*MET* amplification) 2. Cells were categorized as (1) ≤1 copy of *MET*/*CEP7* ; (2) ≥2 copies or ≥3 copies (*MET* amplification)	[48]Engelman et al., 2007
Amplification	*MET* was amplified in NSCLCs from 9 of 43 (21%) patients with acquired TKI resistance but only 2 (3%) tumors from 62 untreated patients	New York Taiwan	aCGH Profiling	Amplification and deletions were defined as segment mean log2 ratios of >1 or less than −1	[46]Bean et al., 2007
Amplification	Among 213 NSCLC patients, increased *MET* copy number identified in 12 patients (5.6%)	Japan	qPCR	*MET* amplification (increased *MET* copy number) was defined as more than three copies	[51]Okuda et al., 2008
Amplification	*MET* amplified in 22 cases (21%) of 106 surgically resected NSCLC patients	France	qPCR	The cut-off value of the normalized ratio established for each pair of reference/target genes (ß-globine and GAPDH for MET); amplified if its normalized ratio is over M +2 SD	[100]Beau-Faller et al., 2008
Amplification	FISH analysis in 435 primary NSCLCs. High *MET* gene copy number (mean ≥5 copies/cell) was observed in 48 cases (*MET*+, 11.1%) including 18 cases (4.1%) with true gene amplification	Italy	FISH	*MET* FISH-positive all cases with mean ≥5 copies per cell	[101]Cappuzzo et al., 2009
Amplification	In TKI-resistant NSCLC patients, *c-MET* amplification in 17.2% (5/29)	China	qPCR	The cut-off value was established as the mean (M)+2 standard deviation (SD) from normal lung tissues of 53 EGFR TKI-naïve patients. A tumor sample was defined as amplification positive if its ratio value was over M+2×SD.	[102]Chen et al., 2009
Amplification	FISH-positive *MET* observed in 16.7%, amplification in 3.9% and high polysomy in 12.8% of 180 resected NSCLCs without TKI treatment (TKI naÏve)	Korea	FISH	Gene amplification (*MET* to *CEP7* ratio 2; >15 copies of the MET signals in >10% of tumor cells; high polysomy (≥40% of cells displaying ≥4 copies of the MET signal)	[45]Go et al., 2010
Amplification	*MET* amplification in 8 (4%) of 183 patients with lung adenocarcinoma	Japan	qPCR	Amplification was defined as a copy number more than 1.31 copies, which was calculated by the mean of the *MET* gene copy number measured plus two times of standard deviation	[56]Onitsuka et al., 2010b
Amplification	*MET* amplification in 4/27 (15%) of TKIs resistant tumor specimens	Boston, MA Hong Kong, China Guangzhou, China	1. qPCR 2. FISH	1. relative to a reference, the Line-1 repetitive element: MET copy number ≥4 (*MET* amplification) 2. Cells were categorized as (1) ≤1 copy of *MET*/*CEP7* ; (2) ≥2 copies or ≥3 copies (*MET* amplification)	[47]Turke et al., 2010
Amplification	*MET* FISH-positive in 11.1% of 380 patients with surgically resected NSCLC (high polysomy, 8.7%; gene amplification, 2.4%)	Korea	FISH	High polysomy (≥4 copies in ≥40% of cells); and gene amplification (presence of tight gene clusters and a ratio of *MET* to chromosome 7 of ≥2 or 15≥ copies of *MET* per cell in ≥10% of cells)	[60]Park et al., 2012
Amplification	Increased *MET* gene copy number occurred in 18.0% of 61 NSCLC tissues	China	qPCR	the cut-off was set to 3 (*MET* gene copy number ≥ 3)	[44]Sun et al., 2013
Overexpression	In primary NSCLC carcinomas from 42 patients: Western blot analysis. MET increased 2 to 10 fold in 25%, HGF overexpressed 10-100 fold compared with adjacent normal tissue. In IHC, MET/HGF homogeneous expression in carcinomas	Italy	1. Western blot 2. IHC	1. The score relative to normal lung tissue of the same patient was as follows: (−), negative samples; (+), detectable expression as in the normal counterpart; (+ +), 2 - 5-fold and (+ + +), more than 10-fold increase	[58]Olivero et al., 1996
Overexpression	Western blot analysis. MET overexpression in 104 patients: 34/47 (72%) in adenocarcinomas, 20/52 (38%) in squamous cell carcinomas. IHC in 104 patients: 56/104 (54%) of NSCLCs consisted of 36/47 (77%) of adenocarcinomas, 19/52 (37%) of squamous cell carcinomas	Japan	1. Western blot 2. IHC	1. Band intensity: (−), undetectable; (+) slight and (++) moderate was 50% level of the positive control; (+++), stronger than positive control	[103]Ichimura et al., 1996
Overexpression	High HGF expression in NSCLC	Pittsburgh, USA	quantitative Western blot	Bio-Rad assay was used to measure protein content of tumor extracts, and results from Western blots were expressed as nanograms of HGF per 40 ug of tumor protein	[59]Siegfried, 1997
Overexpression	In patients with small-sized lung adenocarcinomas, c-MET-positive in 69 of 131 cases (53%)	Japan	IHC	The results were evaluated as positive when bundles of myofibroblasts were stained for c-MET in more than one microscopic area: occasional scattered c-MET-positive cells were considered negative	[104]Tokunou et al., 2001
Overexpression	70% of the 166 primary NSCLC tissues showed strong HGF expression	Canada	IHC	Tumors that showed similar or stronger staining levels as the normal epithelium were scored as high HGF expressors	[105]TSAO et al., 2001
Overexpression	In 88 patients with NSCLCs, 22 carcinomas (25.0%) were intratumoral HGF-positive, and 36 carcinomas (40.9%) were intratumoral c-MET positive	Japan	IHC	1. intratumoral HGF-positive when ≥50% of the tumour cells positively stained for HGF 2. Staining intensity was classified as grade 0 (no staining), grade 1 (weak staining), grade 2 (moderately strong staining), grade 3 (very strong staining), or grade 4 (extremely strong staining). The intratumoral c-MET-positive when the intensity of c-MET-stained tumour cells was greater than grade 1	[57]Masuya et al., 2004
Overexpression	MET strongly expressed in 61% (14/23 cases) of NSCLCs and p-MET in invasive front of NSCLC	Chicago	IHC	Immunohistochemical staining intensity and extent of c-MET using the three-scale scoring system: negative (0), weak (1+), and strong (2+)	[106]Ma et al., 2005
Overexpression	In 130 primary NSCLCs, phospho-c-MET positive in 21.5% (28/130) of cases. MET positive in 74.6% of cases (97/130) and expressed at high levels in 36.1% of cases (47/130). HGF at high levels in 31.5% of cases (41/130)	Japan	IHC	–, complete absence of staining or only focal weak staining; 1+, weak to moderate staining in less than 40% of cancer cells; 2+, weak to moderate staining in at least 40% of cancer cells; 3+, strong staining in at least 10% of cancer cells, among the specimens with weak to moderate staining in at least 40% of cancer cells. c-MET low (− or 1+) or c-MET high (2+ or 3+)	[107]Nakamura et al., 2007
Overexpression	HGF expression was higher in the TKIs resistant specimens	Boston, MA Hong Kong, China Guangzhou, China	IHC	The percentage of cancer cells with positive cytoplasmic and/or membranous staining (0–100%), and the modal intensity of the positively staining cells on a scale from 0 to 4+. The percentage and the intensity were multiplied to give a scoring index ranging from 0 - 400	[47]Turke et al., 2010
Overexpression	Positive expression of HGF in 104 specimens (57%). p-MET 1234/1235 in 12 (7%) specimens of 183 patients with lung adenocarcinoma	Japan	IHC	The score for the positive staining cells were assigned according to the frequency of positive tumor cells (0, none; 1, <1/100; 2, 1/100 to 1/10; 3, 1/10 to 1/3; 4, 1/3 to 2/3; and 5, >2/3). Thereafter, 4 degrees for the intensity score were assigned according to the intensity of the staining (0, none; 1, weak; 2, intermediate; and 3, strong). The intensity score were then added to each other to obtain a total score, which ranged from 0 to 8. A positive expression when the score was 3 to 8.	[56]Onitsuka et al., 2010b
Overexpression	MET IHC-positive in 13.7% of 380 patients with surgically resected NSCLC	Korea	IHC	The intensity score: 0=no appreciable staining in the tumour cells, 1=faint/barely perceptible partial membrane staining, 2=weak to moderate staining of the entire membrane, and 3=strong staining of the entire membrane. The fraction score: 0=less than 5%, 1=from 5% to 25%, 2=from 26% to 50%, 3=from 51% to 75%, and 4=more than 75%. The total score was calculated by multiplying the intensity score and the fraction score, producing a total range of 0-12. For statistical analyses, scores of 0-3 were considered negative, and scores of 4-12 were considered positive	[60]Park et al., 2012
Overexpression	48% of samples (83 of 174) were MET positive.	Poland	IHC	The IHC scoring was done by one pathologist (B.R.A.) using the H-score assessment combining staining intensity (0–4) and the percentage of positive cells (0–100%). Each individual intensity level was multiplied by the percentage of cells, and all values were added to obtain the final IHC score, ranging from 0 to 400. median IHC score for the population as the cutoff point	[108]Dziadziuszko et al., 2012

**Table 3:** Molecular alterations of MET/HGF in human HCC

Alteration	Findings	Population	Technique	Evaluation	Reference
Amplification	0/125 HCC tumors on cytoband 7q31.2	France	SNP genotyping array	1. Chromosome gain: copy number of ≥ ploidy +1 2. High-level amplification: copy number of > ploidy +2	[109]Guichard et al., 2012
Amplification	1/44 (2.3%) cases; 22/44 have aneuploidy of chromosome 7 in resected HCC patients	Tokyo, Japan	FISH	1. *c-MET/CEP7* = 2.0 or higher (gene amplification) 2. mean CEP7 signals of 2.5 or higher per nucleus (chromosome 7 aneusomies)	[43]Kondo et al., 2013
Amplification	High peak frequency 4.5% at cytoband 7q31.2 in 286 HCC patients treated with surgical resection	Seoul, Korea	SNP genotyping array	Copy numbers ≥3 (high-level amplifications)	[42]Wang et al., 2013
Point mutation	Kinase domain: 30% (3/10) childhood HCC 0/65 Adult HCC	Seoul, Korea	PCR-based SSCP	Tumor and corresponding normal DNA from each slide were amplified	[27]Park et al., 1999
Point mutation	0/24 patients after surgical resection	France	Whole-exom sequencing	hg19 reference genome	[109]Guichard et al., 2012
Overexpression	Northern blot analysis. MET overexpression in some cases and underexpression in others. HGF downregulation	N/A	Northern blot	Expression in the tumors compared to the adjacent normal liver	[110]Selden et al., 1994
Overexpression	Competitive RT-PCR. Overexpression in some of the 11 patients by surgical resection. HGF undetectable	Japan	Competitive RT-PCR	Expression in the carcinomatous higher than that in the surrounding non-cancerous tissues	[111]Noguchi et al., 1996
Overexpression	MET overexpression in all 20 HCCs and granular intracytoplasmic positivity for HGF in 9 of 20 HCCs	Italy	IHC	The intensity of HGF protein and c-MET pp plasma-membrane positivity was evaluated as weak (+/++) or strong (+++/++++)	[112]D'ERRICO et al., 1996
Overexpression	Western blot analysis. 52% of 62 patients with MET overexpression, correlating with increased incidence of intrahepatic Metastases and shorter 5-yr OS	Japan	1. Western blot 2. ELISA	Densitometry analysis by the median cutoff value	[61]Ueki et al., 1997
Overexpression	IHC in 86 patients' biopsies. MET overexpression in 20% compared to surrounding hepatic tissue and downregulation in 32%. HGF overexpression in 33% and downregulation in 20%	China	IHC	Arbitrary units based on the intensity of the reaction. 0, no staining; +, weak reactivity: + +, moderate reactivity: + + +, strong reactivity: and + + + +, very strong reactivity, respectively	[62]Kiss et al., 1997
Overexpression	MET protein overexpression in some cases of human HCC	U.S.A	Western blot	Compared with normal livers	[25]Chen et al., 1997
Overexpression	IHC and RT-PCR in 24 HCC. Overexpression of MET in most of the cases. Underexpression of HGF	Italy	RT-PCR	Compared to the surrounding tissues	[63]Tavian et al., 2000
Overexpression	In 30 patients with HCC, MET over-expression in 19 cases and under-expression in 11 cases. HGF overexpression in 10 cases, and underexpression in 20 cases	Japan	Western blot	Each value was obtained from the comparison with the level of ß-actin, and the mean values were calculated from three repeated measures. Expression level compared to non-tumor tissues	[113]Osada et al., 2005
Overexpression	Positive expression of c-MET protein in 27 cancerous regions (27/31) undergoing surgical resection. Higher preoperative concentration of serum HGF in the liver cancer patients	China	1. IHC 2. ELISA	1. intensive positive (+++) when positive cells comprised more than 50% of the total cells; moderately positive (++) when positive cells comprised 16–50%; weakly positive (+) when positive cells comprised 10–15%; and negative (−) when positive cells comprised less than 10% 2. Compared with normal controls	[64]Wu et al., 2006
Overexpression	66.6% of 194 HCC patients with c-MET positive, 5-yr DFS: 61.6% vs 22.75% (c-MET- vs c-MET+)	China	IHC	The extent of positive staining was scored as follows: 0, ≤10%; 1, >10–25%; 2, >25–50%; 3, >50–75%; and 4, >75%. The intensity was scored as follows: 0, negative; 1+, weak; 2+, moderate; and 3+, strong. The final score was obtained by multiplying the extent scores and intensity scores, producing a range from 0 to 12. Scores 9–12 were defined as strong staining pattern (++), scores 0–4 were defined as negative expression (−), and scores 6–8 were defined as intermediate staining pattern (+)	[65]Wang, et al., 2008
Overexpression	C-MET expression in 87.5% HCC patients undergoing hepatic resection. 29 of 40 patients (73%) had increased concentrations of portal HGF	Taiwan	1. IHC 2. EISA	1. Immunoreactivity of c-MET was classified as negative when <10% of the cells stained positively, and positive when ≥10% of the cells stained positively 2. Posthepatectomy portal HGF levels compared to prehepatectomy portal HGF levels	[114]Chau et al., 2008
Overexpression	In 520 total patients, 282 c-MET+ patients (54.2%), correlating with shorter 7-yr OS	China	IHC	Mean area of positive staining as cutoff value, c-MET high, >20% of tumor section	[115]Ke et al., 2009
Overexpression	15/59 (25.4%) cases high level of c-MET expression	Tokyo, Japan	IHC	4-point scoring system: 0 = no staining observed in invasive tumor cells; 1+ = weak, incomplete membrane staining in any proportion of the invasive tumor cells, or weak, complete circumferential membrane staining in fewer than 10% of cells; 2+ = weak but complete membrane staining in at least 10% of cells, or intense complete circumferential membrane staining in 30% or fewer of tumor cells; 3+ = intense complete ircumferential membrane staining in more than 30% of tumor cells. scores 0 and 1+ as c-MET low, and scores 2+ and 3+ as c-MET high	[43]Kondo et al., 2013
Overexpression	High expression of c-MET in 80.6% of 93 HCC patients. No correlation with clinicopathological factors, but correlated with PFS	Italy	IHC	Combination of positive cell count and staining intensity used for scoring. Positive cell count, 0-10%, score 0; 11-25% score 1; 26-50%, score 2; 51-75% score 3; >75%, score 4. Staining intensity: negative score 0; faint yellow, score 1; yellow or deep yellow, score 2; brown or dark brown, score 3. High expression, Score ≥5	[116]Chu et al., 2013

### Prevalence of *MET* gene amplification in cancers

*MET* gene amplifications that result in protein overexpression and constitutive activation of the MET receptor kinase have been reported in NSCLC, gastric cancers and HCC [24]. Variable *MET* gene amplification rates were detected depending on the detection method (e.g., fluorescence *in situ* hybridization [FISH], single-nucleotide polymorphism [SNP] genotyping and quantitative polymerase chain reaction [qPCR]) and the different scoring criteria that define high amplification. In gastric cancer, *MET* gene amplification prevalence varies from 2 to 23% among studies limited by small sample sizes. In one study, a Southern blot using a [α-32P] dCTP-labeled *MET*-H probe (Oncor, Inc., Gaithersburg, MD, USA) detected *MET* amplification in 10% of chemotherapy-naïve primary gastric carcinomas compared with the surrounding normal mucosa [36]. In another study, 21.2% of formalin-fixed, paraffin-embedded (FFPE) primary tumor tissues exhibited *MET* amplification defined as copy number >4 by qPCR [37]. *MET* amplification was detected by FISH in 2% of patients with gastric cancer; in this case, *MET* amplification was defined as a *MET/CEP7* ratio >2 [38]. Several studies with large cohorts correlated *MET* gene amplification with advanced tumor stage and poor prognosis in gastric cancer patients undergoing surgery with or without chemotherapy [39–41]. By contrast, *MET* amplification was barely detectable in patients with HCC who underwent resection: 4.5% by SNP genotyping array (Illumina), defined as a copy number ≥3, and 2.3% by FISH (Abbott Molecular), defined as *MET/CEP7* ≥2.0 [42, 43].

In NSCLC patients with wild-type *EGFR*, *MET* amplification rates ranged between 2.4% and 21% depending on the detection approach and study criteria (Table 2). *MET* amplification was observed in just 3.9% of TKI-treatment-naïve NSCLC patients as measured by FISH (*MET/CEP7* >2), but in 18% of patients when measured by qPCR (*MET* CN ≥3). *MET*-FISH positivity or increased *MET* copy number predicted worse survival [44, 45]. In TKI-resistant NSCLC patients, *MET* is amplified at a higher rate, from 15 to 22%, based on FISH, qPCR and aCGH profiling [46–48]. These findings highlight the need for identification of lung cancer patients with *MET* amplification who will benefit from combination therapy due to primary or acquired resistance, regardless of their *EGFR* status.

### Prevalence of *MET* mutations in cancers

Although *MET* mutations happen rarely in cancers, they may correlate with tumor development. Protein structure alterations, either *via* promotion of receptor dimerization or alteration of catalytic activity, can be attributed to increased kinase activity in MET mutants [15]. Missense mutations were detected in the MET juxtamembrane domain in only 1% of patients with primary gastric cancer using methods such as denaturing HPLC (DHPLC) (Transgenomics) or cold single-strand conformation polymorphism (SSCP; Novex); these mutations may contribute to tumorigenesis [49]. In NSCLC, somatic mutations in the MET juxtamembrane domain result in the deletion of exon 14, which is responsible for recruitment of the E3-ubquitin ligase, Cbl. This leads to the accumulation of abnormally spliced, activated MET unregulated by Cbl-induced degradation. This mutation was associated with elevated MET expression in primary tumors, which was detected in approximately 3% of NSCLC patients [28, 50]. Recently, polymorphisms in the juxtamembrane (R988C and T1010I) and sema (N375S) domains were detected in 1.7 and 4% of NSCLC patients, respectively, by PCR-based sequencing; however, no associations between MET mutations and clinical and pathological NSCLC features were observed [51, 52]. MET mutation was detected only in the kinase domain in 30% of childhood HCC cases by the SSCP method [27].

### Prevalence of MET overexpression in cancers

Immunohistochemistry (IHC), reverse transcriptase PCR (RT-PCR), Western blot and enzyme-linked immunosorbent assay (ELISA) analyses have indicated that MET and HGF levels vary in tumors compared with surrounding normal tissues. MET overexpression was detected *in vivo* in 9.6 to 71% of human gastric carcinomas based on methodology and tissue type (Table 1). Notably, different antibodies that recognize various MET epitopes and domains have shown different membrane and/or cytoplasmic staining intensities by IHC. For example, 46.1% of primary gastric carcinoma patients exhibited cell membrane and cytoplasmic staining in >5% of tumor cells using a C-28 antibody (Santa Cruz Biotechnology) and the Dako ENVISION system [53]. In another study with the same antibody, 63% of patients showed positive MET expression defined as ≥25% of tumor cells with staining intensities of 2+ or 3+ [54]. MET overexpression in gastric cancer ranged from 9.6 to 23.8%, as defined by IHC staining intensities of 2+ or 3+, *via* an SP44 rabbit monoclonal antibody from Ventana Medical System. MET IHC 3+ expression was associated with a shorter OS and PFS, and *MET* gene copy number detected by FISH correlated with MET protein expression detected by IHC in gastric cancer patients [39–41, 55].

The prevalence of HGF or MET in tissues has also been described in NSCLC tumors with wild-type *EGFR*. Positive intratumoral HGF expression was identified in 57% of 104 specimens using an anti-HGF antibody (sc-7949, Santa Cruz) according to the IHC Allred scoring system, and expression was associated with poor OS [56]. Using the same antibody in a separate study, HGF overexpression, in which ≥50% of tumor cells exhibited positive staining, was identified in 25% of 88 patients [57]. Western blot analysis also showed high HGF expression in NSCLC compared with normal lung tissue [58, 59]. HGF overexpression has been implicated in acquired resistance to EGFR inhibitors in patients with EGFR-mutant NSCLC [47]. Notably, the overall rate of MET overexpression measured *via* IHC varied widely from 13.7 to 61% in published studies based on cohorts of various sizes with different types of diseases and scoring systems (Table 2). Still, MET FISH-positive and IHC-positive patients reportedly have significantly shorter survival than MET-negative patients [57, 60].

Similarly, MET overexpression ranged from 20 to 100% in HCCs compared with surrounding normal hepatic tissue (Table 3). Western blot analysis showed MET overexpression in 52% of 62 HCC patients, which correlated with increased intrahepatic metastases and shorter (5-yr) OS [61]. Biopsy IHC analysis of 86 HCC patients revealed 20% with MET overexpression [62], whereas in a separate study employing RT-PCR, most (20/24) patients overexpressed MET [63]. Multiple studies reported that serum HGF concentrations detected *via* ELISA after hepatectomy were higher as compared to normal tissues. Furthermore, HGF levels were correlated with tumor size, node cirrhosis, tumor recurrence or metastases in HCC patients [64].

Multiple studies have demonstrated that MET/HGF is associated with a poor prognosis. A study of 194 HCC patients showed that those with high MET expression with strong staining patterns (++) had significantly shorter 5-year survival than those with low expression with negative staining patterns (−) [65]. Thus, MET may represent a promising target for cancer therapies. MET and HGF alterations are indeed associated with clinical outcome, metastasis, invasion and disease severity in human cancers, and identification of patients with specific alterations may be critical to predict clinical response to targeted therapies. However, based on reported *MET* gene amplification and overexpression prevalence (Tables 1–3), discrepancies clearly result from the use of different detection methods, the number of patients enrolled in a given study, the use of different scoring systems and differences in cancer types. Standardized and optimized methods are needed to identify robust biomarkers that may assist in the selection of MET-positive patients for future clinical trials of MET-targeted therapies.

## BIOMARKER VALIDATION IN MET INHIBITOR CLINICAL TRIALS

Currently, there is no consensus on the optimal platforms to explore the relationship between MET status and drug efficacies in clinical trials. FISH and IHC assays, both traditional and commercialized approaches, require advanced technical skills and experienced experts or pathologists to analyze results. A validated biomarker detection strategy should be developed to identify a predictive or prognostic factor for these therapies. Here, we summarize the correlation between biomarker validation or clinical inclusion criteria and the therapeutic efficacy of selective MET/HGF kinase inhibitors or antibodies in clinical trials of multiple solid tumors, including GC, NSCLC and HCC.

### MET as a therapeutic anti-cancer target

MET signaling dysregulation in cancer is associated with poor patient outcome. In gastric cancer, MET overexpression or amplification is correlated with tumor stage, metastasis, and shorter overall survival (OS) and progression-free survival (PFS). NSCLC patients may develop resistance to EGFR tyrosine kinase inhibitors (EGFR TKIs) *via* EGFR T790M mutation, HGF overexpression or *MET* amplification/overexpression [47, 48, 66]. Of note, *MET* copy number was increased in patients with EGFR TKI-resistant NSCLC compared with TKI-treatment-naïve patients [44–48]. MET activation may compensate for EGFR pathway inhibition *via* activation of the downstream PI3K pathway, and may correlate with acquired resistance to EGFR TKIs in patients with *EGFR*-mutant NSCLC [47].

MET/HGF has been regarded as a promising therapeutic target in cancer treatment, whose gene or protein status may be indicative of patient response to MET-targeted drugs. Numerous preclinical and clinical studies have demonstrated that antibody or small-molecule inhibitors targeting MET or HGF are effective anti-cancer therapies [5, 22, 67].

### Volitinib

Volitinib is a highly selective small molecule, ATP-competitive MET kinase inhibitor being investigated as a monotherapy for *MET*-amplified cancers, such as gastric and lung cancer. Currently, this drug is in clinical development stage, including phase I studies in Australia and China, to test its efficacy against advanced cancers. Other phase I trials seek to test combinations of volitinib and docetaxel in gastric cancer (NCT02252913) and volitinib and gefitinib for EGFR TKI-resistant NSCLC in patients with mutant *EGFR* (NCT02374645).

In preclinical studies, biomarker analysis has shown that *MET*-amplified and MET-overexpressing tumor xenograft models are highly responsive to volitinib as a single agent or in combination with other therapies [68–71]. An *in vivo* study indicated that volitinib selectively inhibited the growth of MET-driven gastric and lung cancer cell line and primary tumor xenografts [68–71]. Anti-tumor efficacy in gastric cancer correlated with *MET* gene amplification/overexpression and high levels of phosphorylated-MET (p-MET). Additionally, the combination of volitinib and docetaxel demonstrated efficacy in a *MET*-amplified gastric cancer cell line and in primary xenograft models [69]. However, correlations between tumor growth inhibition and MET status were less clear in lung cancer compared with gastric cancer. This may be the result of heterogeneity or variation in p-MET or the activation of compensatory pathways (e.g., EGFR, KRAS) in lung cancer [71]. In particular, the combination of volitinib and taxotere had satisfactory efficacy in tumors less responsive to volitinib monotherapy [71]. The *in vivo* efficacy of volitinib was tested in a model of EGFR TKI-resistant NSCLC (HCC872C4R) with acquired *MET* gene amplification. Volitinib with gefitinib produced a synergistic effect compared with volitinib monotherapy, which produced a poor dose response [70]. Therefore, volitinib selectively inhibited tumor growth in a series of human tumor xenograft models with aberrant MET signaling.

MET-FISH was applied to an assessment of *MET* amplification that involved labeling bacterial artificial chromosome (BAC) DNA with Spectrum Red (ENZO) and CEP7-Spectrum Green probe (Vysis Abbott). *MET* amplification was defined as a *MET/CEP7* ratio ≥2 or cluster signals in >10% of tumor cells. MET-IHC (SP44 Ventana Medical System, Roche) was used to detect MET overexpression, which was defined as a staining intensity of 2+ or 3+ (≥10% of tumor cells with membrane or cytoplasmic staining of moderate or strong intensity). MET-FISH or MET-IHC positivity was considered a predictive marker for response to therapy and determined the patient selection criteria. Volitinib demonstrated robust *in vivo* anti-tumor effects on predominantly MET-driven gastric and lung cancers in which the *MET* gene was amplified, while volitinib combined with chemotherapy could produce additional benefits in the treatment of tumors in which MET was a partial driver and where patients exhibited poor responses to volitinib monotherapy [68–71]. In an early clinical evaluation, a volitinib dose-escalation study was performed to evaluate the drug's efficacy against advanced solid tumors, including gastric and lung cancers [72]. In 29 of 35 evaluable patients treated with 100-1000 mg volitinib daily or 300-400 mg twice daily for 21 days, 10% exhibited partial responses (PR), and 59% exhibited stable disease (SD) in at least one post-treatment. Anti-tumor volitinib activities were observed primarily in patients with papillary renal cell carcinoma (PRCC). Furthermore, data suggested that *MET* gene copy number gain could be a biomarker for therapeutic response. The correlation between MET status and clinical efficacy will be further investigated to improve the patient selection criteria for later-stage clinical trials.

### INC280

INC280 (Novartis) is a potent and highly selective MET kinase inhibitor currently being evaluated in early-stage clinical studies. INC280 inhibited cell proliferation, migration and apoptosis in MET-driven tumor cell lines. INC280 suppressed tumor growth *in vivo* in a dose-dependent manner, and was extremely well tolerated, especially in mouse xenograft models of MET-driven glioblastoma and gastric cancer [73, 74].

A phase I clinical study reported stable disease in 24% (8/33) of patients with MET-driven advanced solid tumors, including PRCC, NSCLC, HCC, gastric cancer and others (NCT01324479). INC280 showed preliminary anti-tumor efficacy as a single agent in 50% of patients with *EGFR* wild-type NSCLC with MET dysregulation, as confirmed by FISH (*MET*/centromere ratio ≥2.0 or *MET* gene copy number ≥5) or IHC (MET H-score ≥150 or 50% of tumor cells with a staining intensity of 2+ or 3+) [75].

In MET-positive NSCLC tumors with mutant *EGFR* that were found resistant to EGFR TKIs, a phase Ib/II study of INC280 in combination with gefitinib reported partial responses in 8/46 (17%) of evaluable patients with high MET status (IHC 3+ and/or MET copy number *via* FISH ≥5) (NCT01610336). Overall response rates were 40% in patients with *MET* CN ≥5 and 38% in patients with MET IHC staining intensity of 3+ [76]. Unexpectedly, some NSCLCs with high MET expression (IHC 3+) and amplification exhibited tumor progression following treatment. Whether the platform, reagents and scoring system were appropriate for MET status assessment should be further evaluated in a phase II expansion study. A phase I study of INC280 plus erlotinib in patients with MET-expressing NSCLC (NCT01911507) is ongoing. In HCC, a phase II clinical trial of INC280 as a first-line treatment is currently recruiting patients with tumors that harbor with activated MET pathways (NCT01737827).

### Rilotumumab

Rilotumumab (previously known as AMG 102; Amgen) is a fully human monoclonal HGF antibody that preferentially binds to the mature, active form of the protein [77]. Interaction with rilotumumab prevents HGF from binding to MET, which prevents subsequent signaling [78]. The antibody was tested in preclinical models and in clinical trials in multiple solid tumors, either as a monotherapy or in combination with other chemotherapeutics [79–83]. Based on preclinical data, rilotumumab has shown the anti-tumor activity *in vitro* and *in vivo* studies, [79, 84]. In a phase I clinical trial, rilotumumab monotherapy was deemed safe and well tolerated in 40 patients with refractory advanced solid tumors. A total of 16/23 (70%) evaluable patients achieved stable disease as a best response with PFS from 7.9 to 40 weeks [81].

In a phase II trial (NTC00719550), rilotumumab in combination with the cytotoxic agents epirubicin, cisplatin or capecitabine (ECX) was evaluated in 121 patients with advanced or metastatic gastric or esophageal junction (G/EGJ) cancer. Rilotumumab plus ECX had an anti-tumor efficacy compared to ECX alone, with modestly improved OS (median months, 10.6 *vs*. 8.9; hazard ratio [HR] = 0.70) and PFS (median months, 5.7 *vs*. 4.2; HR = 0.60) [85]. The MET-positive tumors were defined as such when at least 25% of tumor cells demonstrated membrane staining at any intensity using the MET4 monoclonal antibody and the verified MET immunohistochemistry pharmDx kit (Dako North America, Carpinteria, CA, USA). However, rilotumumab plus ECX significantly improved OS in patients with high MET expression compared with ECX alone (10.6 months [95% CI 8.0-13.4] *vs*. 5.7 months [4.2-10.4]). Conversely, MET-negative patients had slightly reduced OS when treated with rilotumumab plus ECX compared with ECX alone (11.1 months [6.9-13.2] *vs*. 11.5 months [5.5-20.5]). A similar trend was observed for PFS [85].

A phase III study to assess the efficacy and safety of rilotumumab in advanced gastric cancer did not confirm the phase II findings and was terminated due to futility results and casualties with an increased number of deaths as compared to chemotherapy [86]. Rilotumumab arm was not superior to placebo arm for OS (median months, 9.6 *vs*. 11.5; HR = 1.37) and PFS (median months, 5.7 *vs*. 5.7; HR = 1.30). OS, PFS and ORR were statistically worse in the rilotumumab arm. Significantly more patients in the placebo arm achieved 12-month OS (49.7% *vs*. 38.4%; *P* = 0.053) and ORR (39.2% *vs*. 30%; *P* = 0.027) compared to the rilotumumab arm. No benefit from treatment with rilotumumab was observed, including patients with higher MET expression, and a higher incidence of fetal adverse events occurred in the rilotumumab arm [86]. We suggest that defining MET positivity as IHC staining in 25% of tumor cells is not stringent enough to establish true MET overexpression, and could lead to negative results in clinical trials. Additionally, rilotumumab was recently reported to be only a partial, not full, HGF antagonist, resulting in HGF-induced MET phosphorylation. This may at least partially explain the poor responses and deaths in rilotumumab clinical trials, and has considerable implications for the use of this therapeutic antibody [87].

### Onartuzumab

Onartuzumab (MetMAb; Roche/Genentech) is a single-armed humanized modified 5D5 anti-MET antibody that binds to the MET sema domain and effectively prevents HGF from binding to MET [88]. Preclinical studies have demonstrated inhibitory effects on glioblastoma U87 and pancreatic BxPC3 and KP4 tumor xenografts, including reduced cell proliferation and motility [88, 89]. In a phase I study, a complete response was observed in a patient with chemotherapy-refractory metastatic gastric cancer with high MET gene polysomy, high MET expression and evidence of autocrine HGF production [90]. In a randomized, double-blind phase II trial in EGFR-unselected NSCLC patients, no increased benefit in PFS (HR = 1.09, *p* = 0.69) or OS (HR = 0.80, *p* = 0.34) was noted in patients receiving onartuzumab plus erlotinib *vs*. placebo plus erlotinib. However, in MET-positive (≥50% of tumor cells with a staining intensity of 2+ or 3+) NSCLC patients (52% of 137 individuals), onartuzumab plus erlotinib was associated with improved PFS (1.5 *vs*. 2.9 months, HR = 0.53, *p* = 0.04) and OS (3.8 *vs*. 12.6 months, HR = 0.37, *p* = 0.002). In contrast, MET-negative patients demonstrated worse OS after treatment with onartuzumab compared with patients treated with erlotinib alone. Despite improved PFS and OS in the MET-positive population, the ORR in this subset was not different from that of the placebo plus erlotinib arm [91], possibly because the inclusion criteria for MET-positive patients may not have been properly defined. A randomized phase III trial in patients with MET-positive advanced NSCLC who were to receive the standard chemotherapy has been terminated because treatment with onartuzumab in combination with erlotinib did not demonstrate a significant difference over erlotinib alone [92].

## CONCLUSIONS

Because MET is involved in the regulation of tumor cell survival and metastasis, a better understanding of individual patient sensitivities to MET inhibitors can help guide clinical trial design. Therefore, the key to success for MET-targeted therapies in human cancers may lie in MET-driven patient selection, which is determined by many factors.

First, an integrated platform that incorporates accurate, validated methods and reagent kits, such as antibodies and substrates for the characterization of MET alterations, will help to improve MET-driven population selection. For example, a phase III trial of onartuzumab showed a lack of efficacy in patients with advanced NSCLC, who were selected for the trial based on MET overexpression detected *via* IHC [91]. However, the IHC detection antibody, SP44, binds the cytoplasmic MET domain, whereas onartuzumab binds to the extracellular semaphorin domain. Reported IHC results vary widely because different antibodies that target distinct MET epitopes are used. It is critical to utilize specific antibodies for the detection of MET expression as a predictive biomarker to refine patient selection in clinical trials.

Next, MET-positive patient selection must also take into consideration of tumor stage and type, along with sample storage conditions, and must incorporate stringent clinical inclusion criteria. A single biomarker or a combination of biomarkers may serve as prognostic factors to fully inform patient selection. For example, in the phase III onartuzumab clinical trial, defining IHC MET positivity as 50% of tumor cells with moderate staining intensity might have resulted in the inclusion of patients with false-positive MET-overexpressing tumors.

In a phase II trial, however, IHC analysis correlated better with significant improvements in OS and PFS compared with FISH analysis. In this study, a *MET*/*CEP7* ratio of two or more or a mean of ≥5 *MET* copies per cell were defined as MET FISH positivity, which may result in the inclusion of patients with no *MET*-amplified tumors [93]. These criteria could explain why NSCLC patients with MET FISH positivity exhibited negative responses to onartuzumab in a phase III trial [92]. Additionally, in rilotumumab studies, defining MET-positive tumors as those having at least 25% of tumor cells with membrane staining at any intensity may also have resulted in a high rate of false positives. These results together strongly suggest that testing for MET levels should include both MET overexpression assessment by IHC and *MET* gene amplification analysis by FISH for improved accuracy and reduced false positive detection rates.

Additionally, MET-driven or MET-positive cancer should be determined by the dominant activation of MET and related oncogenic pathways. MET signaling rarely occurs independently. Other oncogenic pathways, such as EGFR and TGF-β, appear to be involved in tumor progression independent of MET signaling [35]. However, EGFR can also bind with MET and PDGFR, thereby constituting a diversified signal transduction network in cancer cells [94]. In many cancer cases, a combined treatment approach that targets both MET and other functionally redundant RTKs should also be considered.

Thus far, no MET inhibitors or antibodies have been approved for clinical use. Evidence is mounting that fighting MET-driven cancers may depend on many elements, such as the specific drug target, disease stage or type, optimal methods for tumor MET status assessment, and, perhaps most importantly, the identification of robust biomarkers as predictors of patient benefit from MET-targeting therapeutics.
